# Exploring the limits of fold discrimination by structural alignment: A large scale benchmark using decoys of known fold

**DOI:** 10.1016/j.compbiolchem.2011.04.008

**Published:** 2011-06

**Authors:** Siv Midtun Hollup, Michael I. Sadowski, Inge Jonassen, William R. Taylor

**Affiliations:** a Department of Informatics, University of Bergen, Bergen, Norway; b Division of Mathematical Biology, MRC National Institute for Medical Research, London NW71AA, UK; c Computational Biology Unit, University of Bergen, Bergen, Norway

**Keywords:** Protein structure comparison, Protein fold, Protein structure alignment, SAP, DALI, TM-align, TM-score, Decoy model

## Abstract

Protein structure comparison by pairwise alignment is commonly used to identify highly similar substructures in pairs of proteins and provide a measure of structural similarity based on the size and geometric similarity of the match. These scores are routinely applied in analyses of protein fold space under the assumption that high statistical significance is equivalent to a meaningful relationship, however the truth of this assumption has previously been difficult to test since there is a lack of automated methods which do not rely on the same underlying principles. As a resolution to this we present a method based on the use of topological descriptions of global protein structure, providing an independent means to assess the ability of structural alignment to maintain meaningful structural correspondances on a large scale.

Using a large set of decoys of specified global fold we benchmark three widely used methods for structure comparison, SAP, TM-align and DALI, and test the degree to which this assumption is justified for these methods. Application of a topological edit distance measure to provide a scale of the degree of fold change shows that while there is a broad correlation between high structural alignment scores and low edit distances there remain many pairs of highly significant score which differ by core strand swaps and therefore are structurally different on a global level. Possible causes of this problem and its meaning for present assessments of protein fold space are discussed.

## Graphical abstract

**Graphical abstract:**

**Figure:**
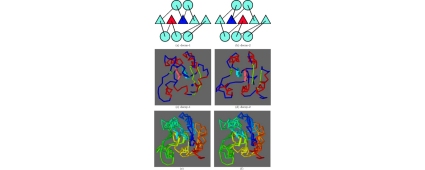

**Highlights:**

► Structure alignment methods are used to assign proteins to fold groups. ► The accuracy of this procedure is difficult to test as the definition of folds can be debated. ► By defining folds topologically and building decoys with defined fold the accuracy of fold assignments can be tested. ► Protein structure alignments decoys of different fold can be assigned a high significance, leading to errors in fold classification. ► This observation can be extended to comparisons between decoy models and real protein structures.

## 1 Introduction

The concept of the “fold” of a protein structure is widely used when discussing broad features of protein structure space, however no universally agreed, consistent definition of the term exists (Harrison et al., 2003; Sippl, 2009; Valas et al., 2009; Sadreyev et al., 2009; Csaba et al., 2009). In general the most common meanings given to the term can be reduced to two: (i) the global structure of the protein and (ii) the structure of a “core” which is broadly conserved through evolution but may accumulate moderate changes and become substantially decorated by embellishments.

Of these two, definition (i) is essentially the meaning of the term as used by structural biologists while definition (ii) is closer to that used by the major classifications of structure, SCOP and CATH (Murzin et al., 1995; Hubbard et al., 1997; Orengo et al., 1997; Greene et al., 2007; Cuff et al., 2008). In describing core structures both classifications make some use of topological description as a means to organise structural data. In this formalism structural details are abstracted out in favour of a definition which describes the arrangement of elements of secondary structure in terms of relative positions and orientations (Richardson, 1977, 1985; Flores et al., 1994; Westhead et al., 1998; Gilbert et al., 1999). For many domains this core topological description may correspond to the entire structure but for others which have experienced substantial embellishments the core may only be a relatively small fraction of the structure or might be obscured by substantial rearrangements such as circular permutations (Andreeva and Murzin, 2006; Krishna and Grishin, 2005; Pan and Bardwell, 2006; Reeves et al., 2006; Grishin, 2001; Harrison et al., 2002; Chaudhuri et al., 2008). Eventually the accumulation of many changes of this sort may lead to adoption of a globally different topology (Grishin, 2001; Peisajovich et al., 2006; Krishna and Grishin, 2005; Taylor, 2007) with a different core, which would be considered a change of fold in either of the two senses defined above.

This evolutionary difficulty in classifying proteins structurally can be avoided if we are prepared to separate the question of evolutionary relatedness from structural similarity, however the question of whether proteins can be meaningfully classified into discrete groups has been debated for some time (Shindyalov and Bourne, 2001; Harrison et al., 2002) based purely on the results of structure comparison. Studies of structural classifications have shown that although there are many broad agreements the existence of many proteins in some groups such as the “superfolds” (Orengo et al., 1994) causes particular difficulty in consistent classification (Hadley and Jones, 1995; Day et al., 2003; Sam et al., 2006; Csaba et al., 2009; Sadowski and Taylor, 2010). Several recent studies in particular have questioned whether the idea of the “fold” as a discrete entity describing protein structure is meaningful (Kolodny et al., 2006; Petrey et al., 2009; Skolnick et al., 2009; Petrey and Honig, 2009), based on the observation of significant structural similarities between proteins with different global structure. However these observations depend crucially on the meaning of statistical significance when applied to pairwise protein structure comparison and whether the rarity of a comparison score compared to the assumed background distribution accurately reflects interesting structural similarity.

Normalization of pairwise scores for structure comparison remains an area of active research (Taylor et al., 2006; Wrabl and Grisin, 2008; Xu and Zhang, 2010). Substructure comparisons are dominated by recurring fragments on a variety of length scales (Taylor and Thornton, 1984; Orengo et al., 1993; Orengo and Thornton, 1993; Salem et al., 1999; Friedberg and Godzik, 2005). Globally the overall tendency of proteins to be compact produces a length-dependency as a consequence of the relationship between radius of gyration and chain length (Maiorov and Crippen, 1994; Reva et al., 1998; Betancourt and Skolnick, 2001; Zhang and Skolnick, 2004). Adding gaps into the comparison complicates matters significantly by adding another parameter which needs to be considered in the background distribution: too few gaps means the alignment will invariably miss significant similarities but allowing too many provides optimization procedures used by structure alignment methods with loopholes to exploit, leading to results of questionable significance such as when a strand and a helix can be aligned to one another.

On this basis it is important to ask whether findings of “significant” similarities between structure pairs necessarily maintain a correspondance between core structures in the face of a significantly similar fragment. To determine how well structure comparison captures this trade-off it is necessary to objectively benchmark methods but this is not a simple issue. Firstly most methods tend to use similar scores: although their implementations and normalizations do vary they are typically based on the same overall framework. Structure classifications attempt to classify global structure using core similarities but there is a risk of circularity since these are largely based on similar methods to those under scrutiny; where differences are found these are often a reflection of subjective choices based on evolutionary and functional relationships which do not necessarily cohere with structural changes (Hadley and Jones, 1995; Day et al., 2003; Theobald and Wuttke, 2006; Sam et al., 2006; Csaba et al., 2009; Sadowski and Taylor, 2010).

To explore this issue we have benchmarked three major structure comparison methods (SAP, TM-align, DALI) using decoys with a three-layer alpha/beta/alpha sandwich structure generated by a structure prediction method capable of exploring a large fraction of topologically distinct folds given an alignment of homologous sequences. Using an edit distance to measure changes on the topological level we distinguish larger from smaller fold changes related to changes in sheet topology and show that all three methods can fail to distinguish strand swaps in the centres of beta sheets at scores which would be deemed of high significance (e.g. TM-score 0.5 or greater (Xu and Zhang, 2010)). Decoy-native comparisons shows the same results and these are used to show that as expected these problems also occur for comparison of real structures.

## 2 Methods

### 2.1 Decoy model generation

Decoy models were built using a method based on the ideal lattice frameworks (“forms”) (Taylor et al., 2008) taken from a periodic table (like) arrangement (Taylor, 2002). The forms describe lattices of secondary structure elements (SSEs) in compact arrangments and guide the assembly of global structure based on known principles of packing observed in a large proportion of proteins.

Decoys were generated from the sequence alignments of four small βα-class proteins: 1coz 126 residues, glycerol-3P cytidyl-transferase; 1di0 148 residues, lumazine synthase; 1f4p 147 residues, flavodoxin (Rossman-like fold); 3chy 128 residues, bacterial chemotaxis Y protein (Rossman-like fold). For each of these probe proteins, we generated ten different multiple alignments, each containing different members of sequences from the same family. Some additional variation was also introduced by the use of two different secondary structure prediction methods. All SSE variations that had sufficient elements to map onto an ideal form were permuted over each form and the resulting folds filtered to remove unfavorable features including left-handed connections in a β-sheet and loop crossings. This resulted in, typically, several thousand folds. These ideal folds were built into α-carbon models, incorporating the variation in secondary structure lengths linked by pseudo-random loop conformations (also of variable lengths taken from the predictions).

The original multiple alignment was then threaded back onto the template fold resulting in some additional shift of the structure away from its idealized origin (Taylor et al., 2006). This was repeated ten times for each fold giving rise to minor structural variation through stochastic components in both the alignment and model construction methods. The threading method also returns a score of how well the alignment fits the model and this was used to rank the models within each distinct fold type. The best model for each fold was retained resulting in a collection of nearly 6000 models. Of these, over 2000 have unique folds with the reduction resulting through the same fold being derived from different starting probe proteins. Interestingly, most of these do not occur in the PDB (Taylor et al., 2009).

### 2.2 Geometry-based comparison

#### 2.2.1 Selected comparison methods

Three comparison methods were used that each have a distinctive feature that allowed us to view the comparison of structures from different aspects as summarized: SAP (Taylor, 1999) an iterated alignment method. (Originally called SSAP (Taylor and Orengo, 1989) and used to generate the CATH database); DALI (Holm and Sander, 1993) substructure matching used widely in searches; TMalign (Zhang and Skolnick, 2005) structural alignment to optimise the TM-score.

Each of these methods have their own differing intrinsic scoring schemes: DALI calculates an estimated Z-score, TM-align uses its TM-score while SAP uses a complex multi-feature score. As well as considering these scores, we will concentrate on a simpler evaluation of the data based on the unweighted RMSD in combination with the number of residues matched which provides a common ground for the different methods.

The raw SAP score we use is not that calculated by the program but is instead a normalization of RMSD and alignment length that was used previously (Taylor, 2006). This is based on a simple square-root function of the number of matched residues damped by a Gaussian function at short lengths to avoid the higher chance similarity of super-secondary structure:

(1) $$r=\sqrt{n}\left(1-\exp-\frac{n^{2}}{\sigma^{2}}\right)\text{,}$$

where *r* is the RMSD value and *n* the number of matched residues. This function has been shown to to be a good approximation of the limit over which comparisons can be considered random. If the Gaussian part of the equation described a normal distribution (half bell curve), then *σ* would correspond to the standard deviation. A value of *σ* = 60 had been used previously but here a slight increase to 70 was used which is a better range for assessing the results obtained below.

#### 2.2.2 Pairwise difference scores

**Fig. 1:**
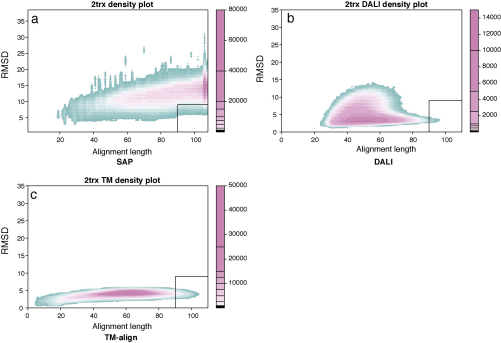
Density plots of structural alignments between decoys and PDB structures. RMSD (*Y*-axis) is plotted against alignment length (*X*-axis) for each comparison. As there are several million data points, the color indicates the number of data points in each region of the plot, with pink indicating high density. DALI does not give as many comparison results as SAP and TM-align, as it only reports a match over a built-in cutoff whereas both TM-align and SAP report a hit no matter how poor it is, giving a wider distribution of data points. The boxed corner (lower right) indicates the region in which good matches that include most of the two structures will be found. (For interpretation of the references to color in this figure legend, the reader is referred to the web version of the article.)

The two values: RMSD (*r*) and number of matched residues (*n*) can be combined into a single value based on a number of possible scoring schemes to give a pairwise difference score. The first score we considered was simply the alignment length divided by the RMSD (*n*/*r*). The second was the alignment length squared over RMSD (*n*^2^/*r*), which will favour longer alignments. A third score was devised to give prominence to those pairs that have a similarity lying in the boxed region in Fig. 1. Rather than use a box, we formulated a partition curve as the square-root of *n* combined with an exponential switch-function (replacing the less flexible Gaussian function in Eq. (1):

(2) $$r=a\sqrt{n}\left(1-\frac{1}{\left(1+\exp((bN-n+c\right)}/d\right)\text{,}$$

where *n* is the length of the match and *N* is the maximum number of matched residues. The parameter *a* scales the height of the curve and *d* controls the steepness of the function at its switch point which occurs at length *bN* + *c*.

This function generates a smooth box-like boundary that can also be used as the basis for a score by finding value of *a* that causes the curve to pass through any given point in the plot. This is similar to the Gaussian damped square-root function used previously (Taylor, 2006) (Eq. (1)).

### 2.3 Topology based comparison

#### 2.3.1 Topology string definition

The topology strings that define the folds were derived from a simplified coordinate system that describes the chain path through the secondary structure lattice (Johannissen and Taylor, 2004; Taylor and Aszódi, 2005; Taylor et al., 2009). The letters “A”, “B” and “C” designate three secondary structure layers (α, β, α, respectively) and a number specifies the relative position of the SSE in the layer with the remaining dimension requiring only two values, “+” or “−”, to indicate direction. The first SSE to enter a layer is assigned position 0 and the first strand in the sheet takes the positive orientation, giving “+B+0” in the string. The first α-helix then sets the top/bottom orientation by assigning its layer as “A”.

It should be noted that this description is slightly more general than a straightforward list of the nodes occupied by the chain as the relative numbering in the helical layers (“A” and “C”) admits the possibility of unoccupied nodes between two occupied positions so that helices +1 and +2 in layer A, for example, are not necessarily spatially adjacent in the structure. In general, the helices are free to ‘shift’ within a their layer but not to swap positions. This does not apply to a β-sheet layer as no gaps are allowed within the sheet. In practice this means that two structures with the same topology string can have differing twists, bends and shears in the location of their secondary structure positions as well as differing lengths for each SSE. The strings thus capture the topological, as distinct from the geometric, nature of the match.

#### 2.3.2 True/false fold match partition

As well as providing a score, the partition curve (Eq. (2)) can be scaled to find the best separation between matches that have the same fold (T, true) and matches with different folds (F, false). True matches have a topological distance of zero, false matches any other value.

True matches below the curve are designated “positive” (P) and above “negative” (N). “TP” then represents the number of true positives and “TN” the number of true negatives while the false matches below the curve are false positives (FP). The simple scoring scheme:

(3) $$s=100\frac{\left(\text{TP}-w\text{FP}\right)}{\left(\text{TP}+\text{FN}\right)}$$

provides a score (*s*) that does not use the TN count as this can be very large and variable between different data sets and is sometimes artificially truncated. The value of *s* was maximised by variation of the parameters *a*, *b*, *c*, *d* in Eq. (2). A weighting factor, *w*, of 0.5 was applied to the FPs giving more weight to the less abundant TP data. The value of this factor determines how many TPs lie below the optimised curve and a value of 0.5 resulted in a curve that enclosed typically 80% of the TPs. The exact scaling of the curve (the value of *a*) is less important than its shape as this is used to rank the matches and should be based on a fit that encloses a large fraction of true matches.

## 3 Results

### 3.1 Decoy/decoy comparisons

#### 3.1.1 Overview of pair comparisons

The 6000 decoy models that resulted from the construction protocol outlined in Section 2 were compared to each other using SAP, TM-align and DALI. To illustrate the differing behaviours of these comparison methods we have taken the pooled decoy-native comparison data and plotted RMSD against the number of matched residues (Fig. 1). The two sub-structure based methods (DALI and TMalign) both return low RMSD values but at the expense of the number of aligned residues. When assessing databank hits or model/native similarity, towards which the two methods are (respectively) directed, this is completely appropriate behaviour. By contrast, the SAP methods returns more matched residues at the expense of RMSD. The SAP method employs what is now known as a global–local alignment algorithm in which terminal indels are not penalised but otherwise it attempts to align as much of the two structures as possible based on semi-local structural similarity rather than overall RMSD.

Since we are interested in how match scores relate to global structural features, only pairs for which the alignment covered the majority of both proteins were considered. This requirement is roughly indicated on the plots by a box in the lower right corner which encloses alignments that have both more than 90 matched residues (typically 80–90% of the shorter protein) with a RMSD under 9 Å. It might appear that very few comparisons lie in this region but as the plots include several million comparisons, even the blue density comprises many pairs.

**Fig. 2:**
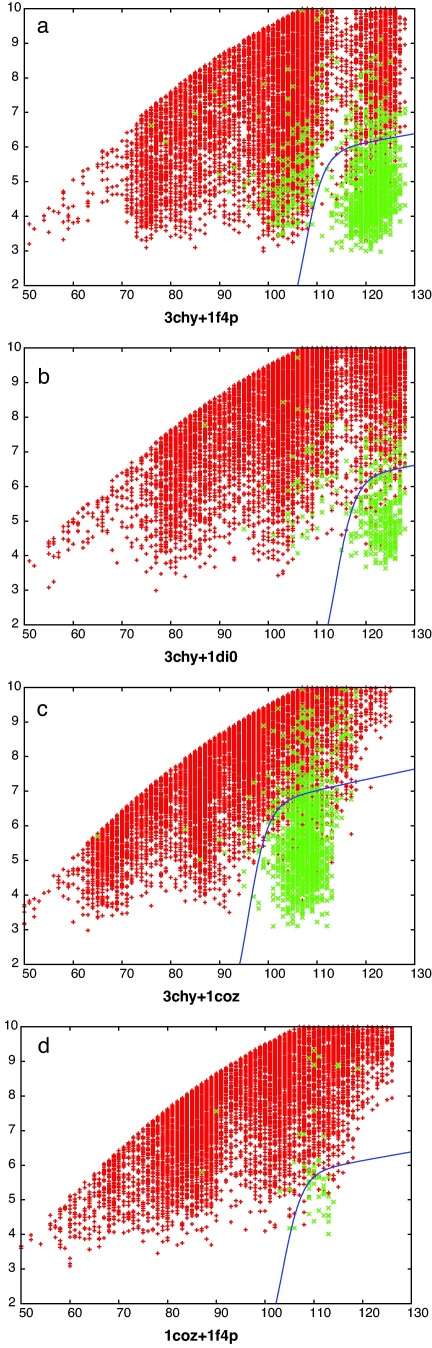
SAP-based decoy/decoy comparisons. Each point marks the result of the comparison of a pair of decoy models, plotted as the number of matched residues (*X*-axis) against their RMSD value (*Y*-axis). The green points have identical topology strings (same fold) whereas the red points have different strings. The upper edge to the red points marks the limit beyond which comparisons were not considered. (Only up to a maximum of 10,000 of these are plotted for clarity). The blue curve is the optimised function that partitions the red and green points. (For interpretation of the references to color in this figure legend, the reader is referred to the web version of the article.)

**Fig. 3:**
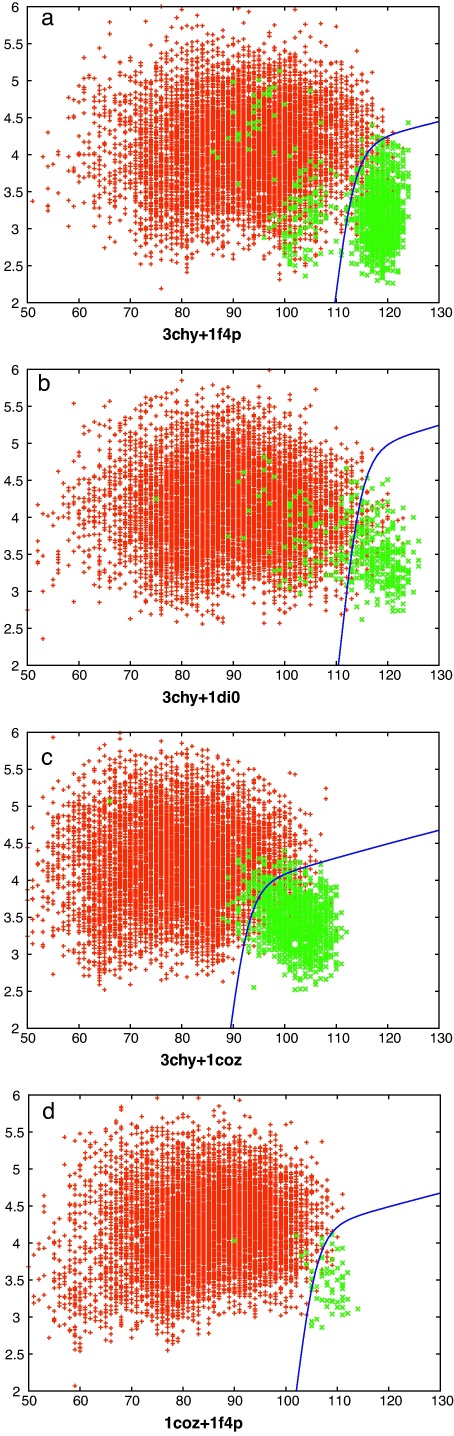
TM-align-based decoy/decoy comparisons. See legend to Fig. 2.

The comparison of the models that have resulted from any pair of starting probe proteins generate alignments that, clearly, cannot exceed the length of the shorter protein. Conveniently, both 3chy and 1coz have almost the same length which means that the comparisons of both these sets with each other and the two larger proteins 1f4p and 1di0 all have the same maximum length (126 residues). This provides five sets of comparisons that can be analysed before the complicating factor of length variation need be considered. However of these, one set contained only two common folds and was omitted. The four remaining sets of comparisons for both comparison methods (SAP and TM-align) are shown in Figs. 2 and 3, respectively. In these plots, it is clear that comparisons between decoys with identical folds (green) lie towards the lower right corner of the plot, having a low RMSD over close to the maximum number of residues aligned, compared to the body of points (red) where the folds differ.

What is also clear from these plots is that there are a significant number of matching folds that fall well away from the lower right corner, especially with the 3chy + 1f4p decoy sets (Figs. 2a and 3a), and more so with the SAP comparison method. This is the consequence of the structural latitude between proteins with identical topology: different element lengths, positions and packing angles can all contribute to large geometric distances for the same topology.

Also of note is the observation that a considerable number of pairs with different folds lie towards the bottom right corner, apparently having a quality of match that is comparable to other pairs with the same fold. These pairs of structures may include those that have “easy” fold transitions in which, say, a loop region has shifted towards an α-helix or β-strand conformation. To investigate this requires that the quality of match is quantified and the comparisons ranked and to do this we used the scores described in Section 2 that transform the RMSD (*r*) and number of matched residues (*n*) into a single value, along with the intrinsic scores calculated by each comparison method.

#### 3.1.2 Finding the best partition curve

**Fig. 4:**
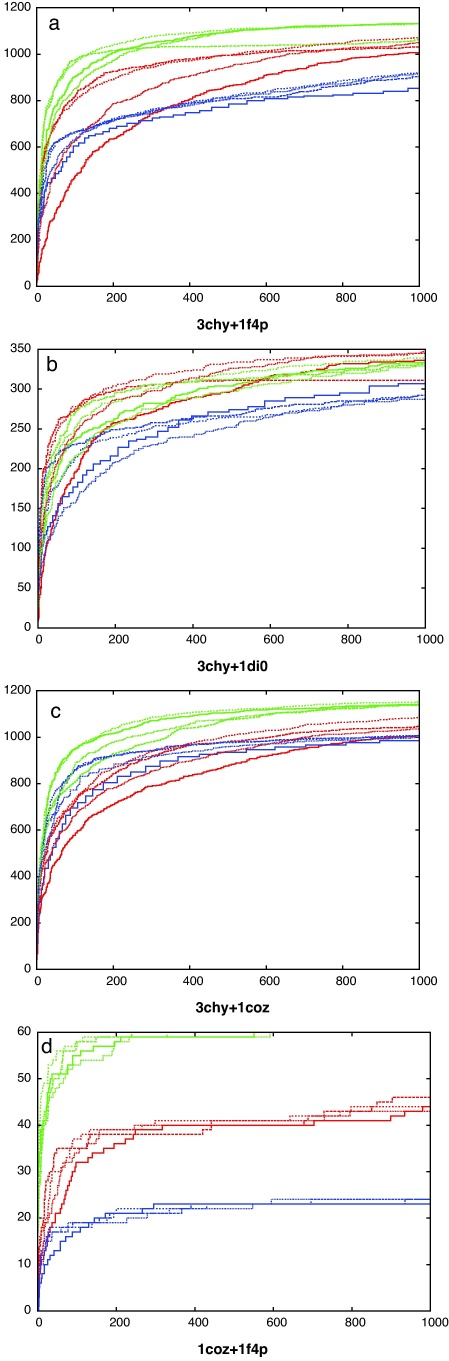
Decoy fold discrimination. Decoy comparisons ranked by different methods: SAP (red), TM-align (green) and DALI (blue), using the various scores described in Section 2. As each ranking is traversed, the number of true topological matches (*Y*-axis) is plotted against the number of false matches (*X*-axis). The best discrimination is attained by methods that have curves approaching the top-left corner. (i.e. most true hits with least false hits). Over the four data sets the renormalised TM-align score gives the best consensus performance (bold green curve). (For interpretation of the references to color in this figure legend, the reader is referred to the web version of the article.)

The optimal form for the partition curve that encloses the lower-right corner of good matches in Figs. 2 and 3 (blue line) was found by varying the four parameters of the curve (*a*, *b*, *c*, *d* in Eq. (2)) to optimise the value of *s* in Eq. (3). The absolute scale (*a*) of this curve is not important as it is used only to score each comparison by the value of *a* that causes the curve to pass through each data point on the plots. Ranked by this value, the data can be re-plotted showing the growth of true matches (same fold) against the growth of false matches (different folds) as the ranked list is traversed. These curves are compared for each of the two comparison methods along with the fold ranking based on their own scoring values and the simpler reciprocal RMSD-based score described in Section 2 (Fig. 4).

It was found that the raw SAP score is a poor basis for discrimination, largely due to the incorporation of many shorter alignments. When re-ranked by the partition curve, discrimination improves markedly as these shorter alignments are now down-weighted. The raw TM-align score provides a good discrimination and improves only slightly with re-ranking based on the partition curve (bold green curves in Fig. 4). Although this TM-align-based ranking was never the best in any individual comparison data-set, it had a good enough overall performance to allow us to avoid the use of separate scoring schemes and methods for each comparison dataset and will be used in the analysis of decoy matches below.

### 3.2 Decoy to native comparisons

Using topology strings derived by applying a match procedure to the SCOP database (Taylor, 2002; MacDonald et al., 2010) we assessed whether comparisons between decoy structures and real proteins suffered from the same problems.

We divided our results decoys with topologies which were not found in any known structure (Taylor et al., 2009) by searching the set of all substrings derived from form fits to the SCOP database (Section 2) for the string corresponding to the decoy. This allows a decoy to be classified as a decoy of unknown fold (DUF) or a decoy with observed fold (DOF).

In the current analysis, the category of matches that is most interesting are those pairs in which there is a good α-carbon correspondence but with different folds (defined as “false positives” in the previous section). If a decoy model has a good match to a known protein at the residue level (positive) and the decoy does not correspond topologically to any known fold (false), then the match must be a false positive since no known domain has a corresponding topology. This is not to say that the matches between DOFs and real proteins with good scores are necessarily correct, but it provides a basis for an initial ranking.

#### 3.2.1 Analysis of the comparisons

**Fig. 5:**
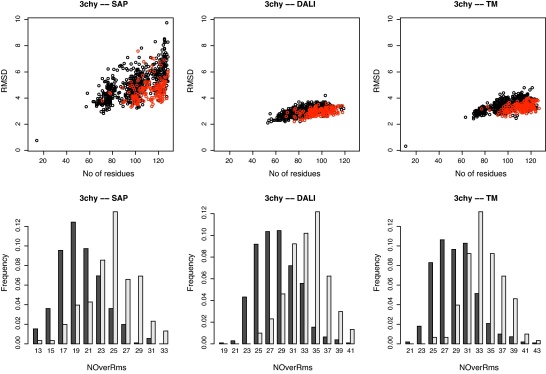
Distribution of DOF and DUF matches to known structures. Upper panels plot the comparison of decoy models with native structures as RMSD (*r*, *Y*-axis) against the number of matched residues (*n*, *X*-axis). To reduce the number of points, only the best match is plotted for each decoy. If the decoy model does not have a known fold (DUF) it is marked by a black dot while those with a known fold (DOF) are red dots. Results are plotted for the three comparison methods: SAP, DALI and TM-align using the dataset of decoys derived from the protein 3chy. The lower panels show these data plotted as frequency histograms based on the value of *n*/*r* with DUFs as filled columns and DOFs open columns. (For interpretation of the references to color in this figure legend, the reader is referred to the web version of the article.)

The 6000 decoy models were matched against 3700 domains from a high-resolution subset of SCOP10 (see Section 2 for details) using the three comparison methods (SAP, TM-align and DALI). As an initial survey, we considered only the models derived from one dataset with the simplest score, alignment length (*N*) divided by RMSD (*R*) which, from the analysis of the decoy/decoy comparisons, had performed reasonably well. (Fig. 5). In all the plots there is a distinct population of known folds at high score values (good matches) but also considerable overlap of these with unknown folds towards lower score values. The *N*^2^/*R* score produced a similar picture. (Data not shown.)

**Fig. 6:**
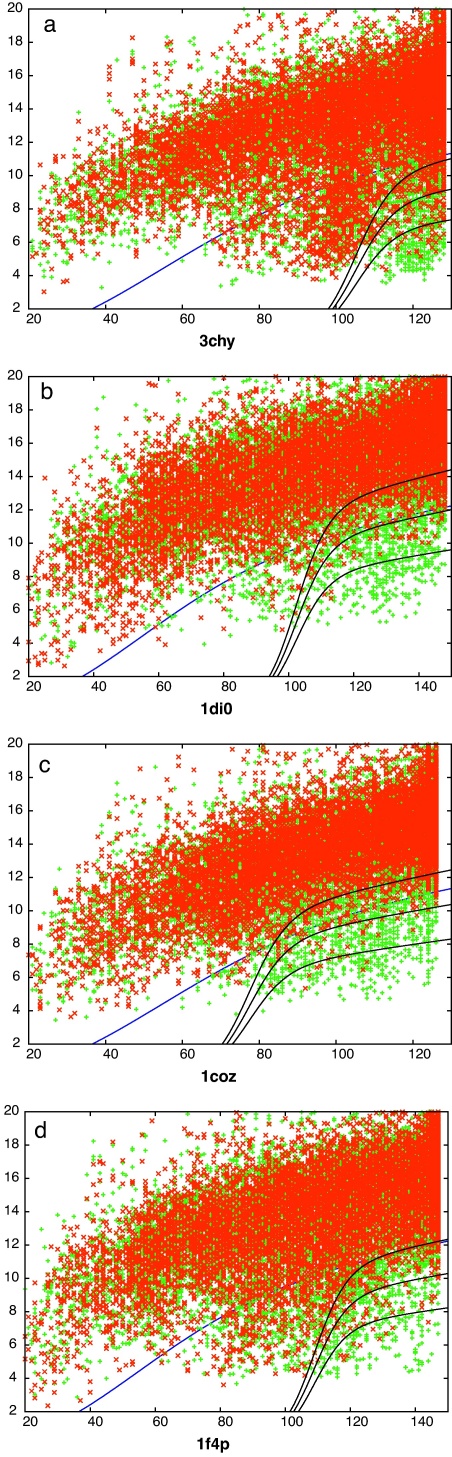
Known folds matched to decoys using SAP. RMSD (*Y*-axis) is plotted against the number of aligned residues (*X*-axis) for proteins matching DOFs (green) and DUFs (red). The blue curve marks the upper limit for meaningful comparison and the black line is the optimised partition function with its ±20 percentiles either side. (For interpretation of the references to color in this figure legend, the reader is referred to the web version of the article.)

**Fig. 7:**
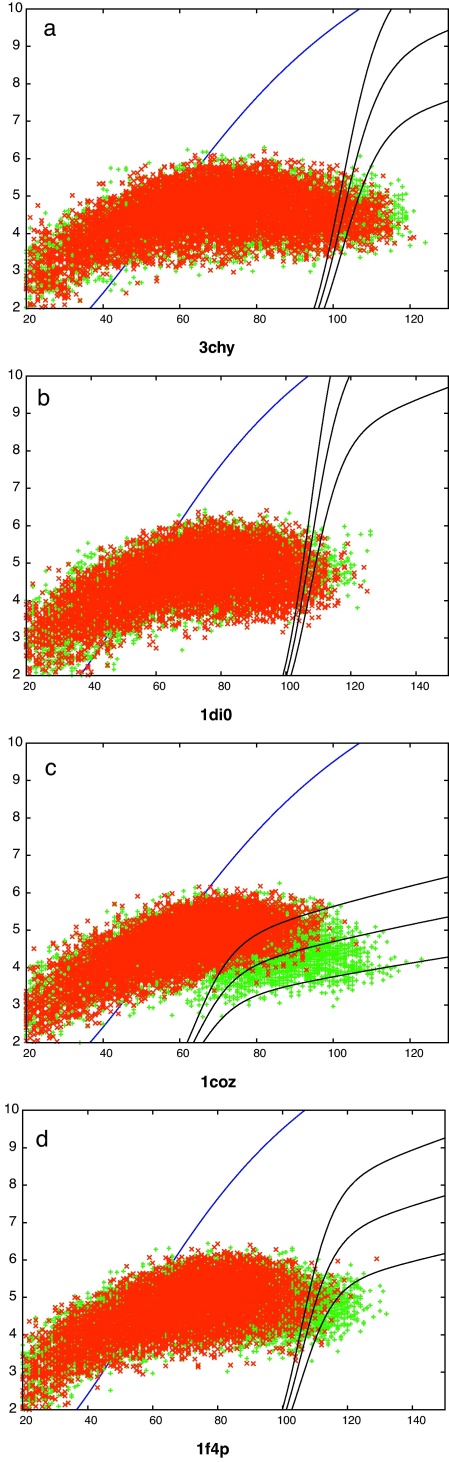
Known folds matched to decoys using TM-align. See legend to Fig. 6.

**Fig. 8:**
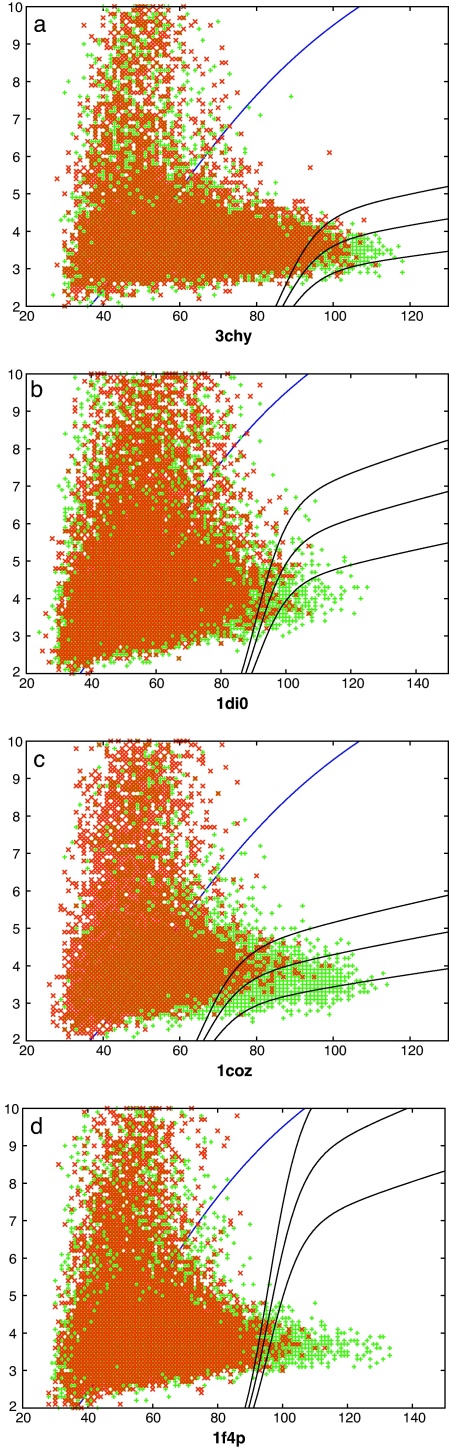
Known folds matched to decoys using DALI. See legend to Fig. 6.

Unlike the decoy/decoy comparisons which all involve structures of a similar size, the decoy/protein comparisons involve a larger size range for the known structures, giving rise to many spurious partial matches with higher RMSD and shorter lengths. Despite this, there is a reasonable number of good matches that involve DOFs repairing with their fold partners or a homologue and also a number of DUFs attain a good match. In the following analysis we turn to the 2D plots of RMSD against length (Figs. 6–8) to find the best scoring scheme to distinguish correct DOF matches from the background and then use this measure to identify DUFs with similarly high-scoring RMSD matches to proteins.

#### 3.2.2 Fold rankings

**Fig. 9:**
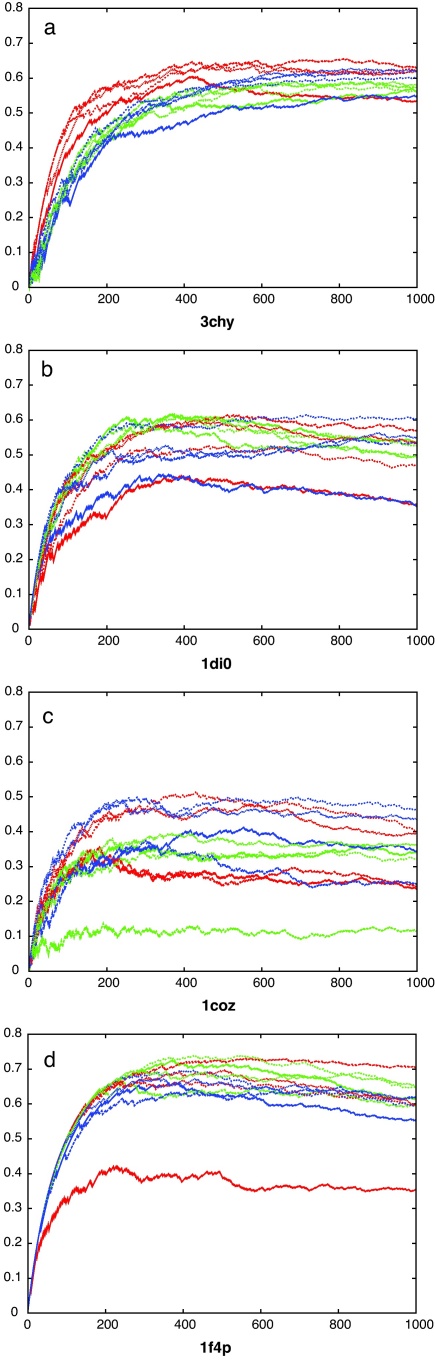
Performance curves for methods and metrics. Each comparison of a decoy and a protein is scored by each method and scoring scheme combination (SAP = red, TM-align = green and DALI = blue) and the pairs placed in rank order (*X*-axis). For each position in the ranking, the value (TP–FP)/(TP + FP + 100) is plotted (*Y*-axis) as a curve. Unlike a ROC plot, these curves can drop if the number of FP counts increases faster than the TP counts. The thicker red line marks the renormalised SAP score which, except for the 1coz dataset, performed well. (For interpretation of the references to color in this figure legend, the reader is referred to the web version of the article.)

The decoy/known datasets are dominated by the large numbers of semi-random background comparisons, making it difficult to distinguish the performance of the various scoring schemes by a simple ROC-like analysis. Following Eq. (3), we avoided not only the TN counts but also the FN counts and used the simple score of: (TP–FP)/(TP + FP + 100). The value of 100 in the denominator was included only to damp fluctuations at low counts. In all but one of the data sets, the SAP comparisons re-scored by the partition curve (Eq. (2)) performs well (Fig. 9). The exception is the dataset derived from the 1coz probe which visually has a good separation of DOFs from the background (Fig. 6) but many true matches are lost to the TN region through having full length alignments with a high RMSD. Rather than employ a variety of scores, the re-scored SAP comparison was taken as a reasonable consensus and the results discussed below are based on this.

### 3.3 Example Comparisons

As much of the earlier sections have been focused on relatively abstract representations and discussion of protein folds, in this section, we provide examples of the types of comparison that is being evaluated, including decoy-to-decoy comparisons and decoy-to-native comparisons.

In all of these comparisons, we have focused on changes that can unambiguously be described as a change in fold. These do not involve any subtle change in angle between secondary structure elements or the addition and deletion of minor edge components (embellishments)—all incorporate an exchange in position between two core β-strand s. From the earliest studies (Richardson, 1977) to later analyses (Ruczinski et al., 2002; Grainger et al., 2010), such changes have been considered to be a change of fold or topology^1^.

In the context of the small βα-proteins considered in this work, most have a five-stranded β-sheet. If we do not consider edge strands, then we expect to see only the permutations of the three central strand positions. Labelling these 1, 2, and 3, gives only three situations: 2,1,3; 1,3,2 and 3,2,1. Note that these are positions in the sheet not strands in the sequence so the three types of strand swap can be found underlying a wider variety of topologies. If the structure is oriented with the first β-strand in the sequence approaching and the first helix above the sheet, then the two single strand swaps can be distinguished.

### 3.4 Decoy-to-decoy comparisons

**Fig. 10:**
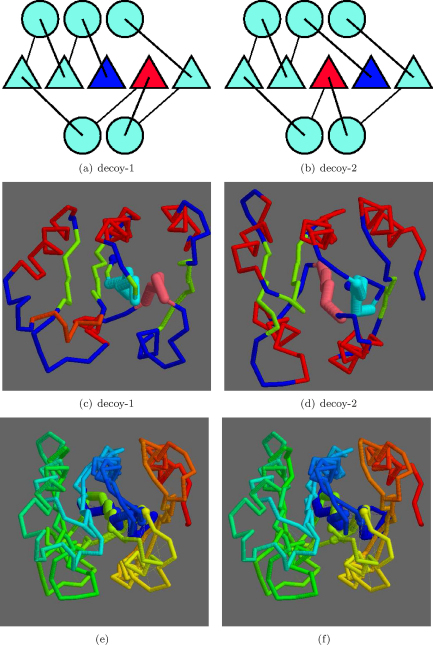
Decoy/decoy 2,3-strand swap. The topologies of the two decoys are shown in parts a and b with the swapped strand positions marked blue (more amino) and red (more carboxy). The corresponding α-carbon models are shown in c and d, respectively, with the swapped strands in light-blue and pink. Other strands are green and helices are red. (The amino terminus is marked by a blue ball). Parts e and f show the superposed models coloured blue from amino to red at the carboxy terminus as a stereo pair. The swapped strands are drawn more thickly. (For interpretation of the references to color in this figure legend, the reader is referred to the web version of the article.)

The comparison data from Figs. 2 and 3 were ranked on their TM-score and each was examined in turn for topologies that differed by swapped core strands. Adjacent strand swaps were common, with swaps involving the terminal strand being more frequent—presumably because it involves the shift of one less connecting loop. The highest scoring pair is shown in Fig. 10 which involves a swap of the 2,3 positions in the sheet. The TM-score for this pair was 0.59 and the SAP comparison gave an RMSD of 5.07 Å over 113 residues.

**Fig. 11:**
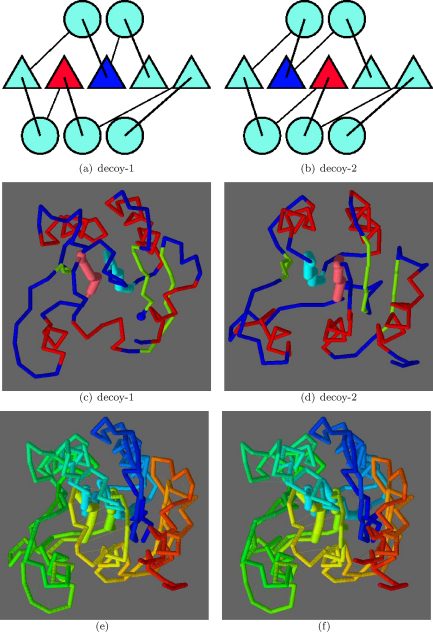
Decoy/decoy 1,2-strand swap. As in Fig. 10, the topologies are shown in parts a and b with swapped positions blue (amino) and red (carboxy). Corresponding α-carbon models are shown in c and d, respectively, with the swapped strands in light-blue and pink. (Other strands green, helices red and the N-terminus a blue ball). Parts e and f show the superposed models coloured blue (amino) to red (carboxy) with swapped strands are drawn more thickly. (For interpretation of the references to color in this figure legend, the reader is referred to the web version of the article.)

**Fig. 12:**
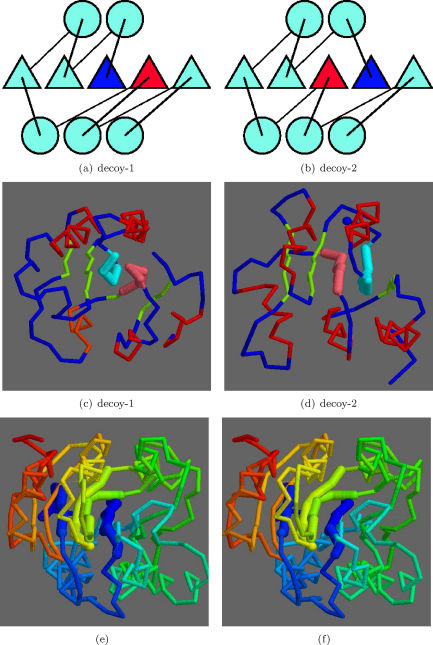
Decoy/decoy 2,3-strand swap (alternative match). Parts a and b show the topologies with swapped positions blue (N) and red (C) as in Fig. 10. Corresponding α-carbon models are shown in c and d, respectively, with the swapped strands in light-blue and pink. (Other strands green, helices red and N-terminal blue ball). Stereo pair e and f show the superposed models coloured blue (N) to red (C) with swapped strands thicker. (For interpretation of the references to color in this figure legend, the reader is referred to the web version of the article.)

At rank 3, a strand swap involving the 1,2 positions was seen (Fig. 11) with TM-score 0.58 and a SAP RMSD of 4.77/110 (Å/res.). As with the 2,3-swap, the bulk of the two models was matched with just the two swapped strands displaced. An alternative to this type of superposition was encountered at rank 13 (Fig. 12), for a 2,3-swap, where the two more carboxy located strands (pink in parts c and d) retained a closer match, with the amino terminal strands (blue) being excluded from the match. The drop in TM-score to 0.54 is only slight with a corresponding drop in the number of residues matched by SAP to 4.8/85 (Å/res.).

### 3.5 Decoy-to-known comparisons

**Fig. 13:**
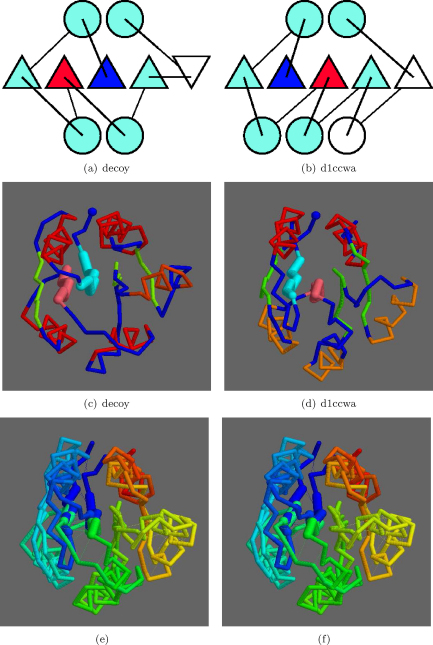
Native/decoy 1,2-strand swap. As in Fig. 10, the topologies are shown in a and b with swapped positions blue (amino) and red (carboxy). The secondary structure elements (SSEs) in white have no match. Corresponding α-carbon models are shown in c and d with the swapped strands in light-blue and pink. Helices are coloured red in the decoy model but red on the top layer and orange on the lower layer for the native structure. (Other strands green and the N-terminus a blue ball). Stereo pair e and f show the superposed models coloured blue (N) to red (C) with swapped strands are drawn more thickly. (For interpretation of the references to color in this figure legend, the reader is referred to the web version of the article.)

The data from Figs. 6–8 were ranked by TM-score and the best match of a native structure to a decoy with different topology was to the Chey-like protein with PDB code: 1ccw (ASTRAL domain: d1ccwa_). The decoy model also had a flipped edge strand and an off-lattice α-helix that formed an bridging link to this antiparallel strand, yet despite these additional topological differences, the match had a good TM-score of 0.66 and a SAP match of 4.65 Å over 125 matched α-carbon atoms. The superposition showed a good overall match, with the swapped strands lying on top of each other (Fig. 13). The swapped strands lie in core positions 2 and 3 in the numbering defined above.

**Fig. 14:**
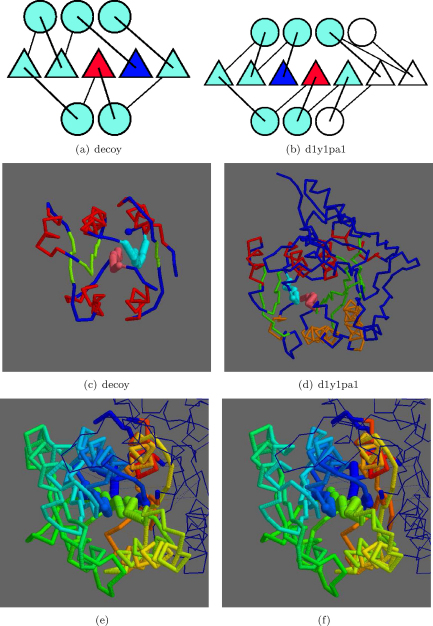
Native/decoy 2,3-strand swap. As in Fig. 13, topologies are shown in a and b with swapped positions blue (N) and red (C). The SSEs in white have no match. Corresponding α-carbon models are shown in c and d, respectively, with the swapped strands in light-blue and pink. Other strands are green, helices red (red/orange in native) and the N-terminus a blue ball. Stereo pair e and f show the superposed models coloured blue (amino) to red (carboxy) with swapped strands are drawn more thickly. Parts of the native structure that are not involved in the match are drawn as a fine trace. (For interpretation of the references to color in this figure legend, the reader is referred to the web version of the article.)

Native structures have a wider range of size than the decoys (generally on the larger side) which means that the superpositons do not always completely coincide. This is seen where a decoy with topology corresponding to that seen in Figs. 10b and 12b is matched within the large protein with PDB code: 1y1p, specifically the domain defined by the ASTRAL domain with code: d1y1pa1. Within this domain, the secondary structures lying on a regular lattice were automatically defined as a 3-7-4 form (a layer of 3 helices over a 7-stranded sheet over a layer of 4 helices) with the topology of an extended Rossman fold (Fig. 14). The swapped strands lie deep in the core of the native structure and by reference to the decoy fold, involve positions 1 and 2 (as defined above). With a large deletion in the native structure, the corresponding parts of the native and decoy structures match well, with a TM-score of 0.61 and a SAP match of 6.96/126 (Å/res.). The superposed register of the two β-sheet s is similar to that seen in Fig. 12.

**Fig. 15:**
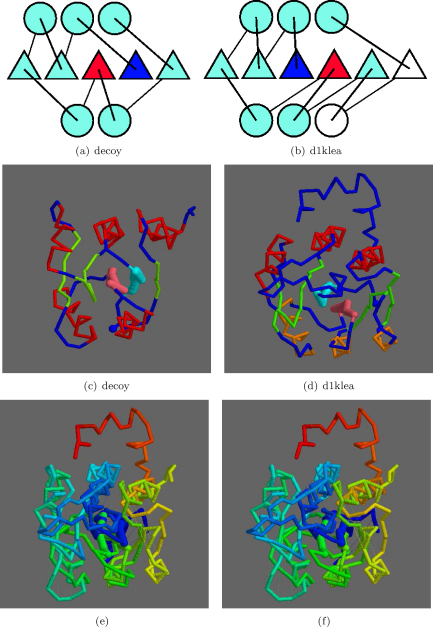
Native/decoy 2,3-strand swap. The topologies are shown in parts a and b with swapped positions blue (N) and red (C), as in Fig. 13. (SSEs in white have no match). Corresponding α-carbon models are shown in c and d, respectively, with the swapped strands in light-blue and pink. Helices are coloured red (decoy) and red/orange (native) with other strands green and the N-terminus a blue ball. Stereo pairs e and f show the superposed models coloured blue (N) to red (C) with swapped strands are drawn more thickly. (For interpretation of the references to color in this figure legend, the reader is referred to the web version of the article.)

An equivalent match of the same decoy fold to a “classic” Rossmann fold is found at rank 5 to the smaller protein: 1kle (ASTRAL, d1klea). The superposition of these structures has a good overall match with the two swapped strands lying over each other as in Fig. 15. The match has a good TM-score of 0.60 but the less good SAP match of 7.71 Å over 128 positions. The larger RMSD value appears to come from two helices that have a match shifted by one turn.

## 4 Discussion

For the purpose of fold discrimination, the results for the decoy/decoy matches and, to a lesser extent, for the decoy/native matches showed that there is little to choose between different comparison methods or scoring schemes, with only a few that performed markedly poorly. However, there was a general trend for those that calculate more extensive matches, covering a greater proportion of the chain, to be better at retaining the fold identity. Where the method is able to discard a significant fraction of the structures, such as DALI, and to a lesser extent TM-align, then key features of the fold can be lost. This was especially true for the decoy/native comparisons and is likely to be even more pronounced for native/native comparisons.

When the folds were ranked on the method and score that provided the best fold discrimination, it was possible to look at examples where good residue-level comparisons were obtained but with a topological mismatch. The most significant changes in terms of topology were selected in which pairs of β-strand s had “swapped” positions in the core of the proteins. Close examination of the superposition of these pairs showed that good quality could be maintained in the overall match despite the shift between the two swapped strands. This was achieved by keeping the two strands sitting in their swapped positions, giving the added RMSD associated with their strand separation (5 Å over several residues). Alternatively, discounting the contribution of one pair by an alignment indel allows an intercolation of strand positions in which each pair is displaced by less than a strand separation.

The matches observed by these topologically different structures were of a sufficiently high quality that most would be accepted as significant by the three structure comparison methods used: A TM-score over 0.5, a DALI Z-score over 5 and a normalised SAP score under 1. For one-off comparisons, topological errors would be found by visual inspection but where the process is completely automatic, as in the assessment of a large number of predicted models or in the exploration of protein fold-space, then a failure to recognise topological differences becomes important.

## Footnotes

1 We use the terms fold and topology to refer to the same property which in our definition is any difference that involves a change in direction of position of a SSE on the ideal lattice. This is equivalent to stating that there is no combination of anisotropic shear, twist or scale operations that could be applied to convert one structure into the other, which captures the essence of the more mathematical use of the term “topology”.

## References

[bib0005] Andreeva A., Murzin A.G. (2006). Evolution of protein fold in the presence of functional constraints. Curr. Opin. Struct. Biol..

[bib0010] Betancourt M., Skolnick J. (2001). Universal similarity measure for comparing protein structures. Biopolymers.

[bib0015] Chaudhuri I., Soding J., AN L. (2008). Evolution of the beta-propeller fold. Protein Struct. Funct. Genet..

[bib0020] Csaba G., Birzele F., Zimmer R. (2009). Systematic comparison of scop and cath: a new gold standard for protein structure analysis. BMC Struct. Biol..

[bib0025] Cuff A., Sillitoe I., Lewis T., Redfern O., Garratt R., Thornton J., Orengo C. (2008). The cath classification revisited—architectures reviews and new ways to characterize structural divergence in superfamilies. Nucleic Acids Res..

[bib0030] Day R., Beck D., Armen R.V.D. (2003). A consensus view of fold space: combining scop, cath and the dali domain dictionary. Protein Sci..

[bib0035] Flores T.P., Moss D.S., Thornton J.M. (1994). An algorithm for automatically generating protein topology cartoons. Protein Eng..

[bib0040] Friedberg I., Godzik A. (2005). Connecting the protein structure universe by using sparse recurring fragments. Structure.

[bib0045] Gilbert D., Westhead D., Nagano N., Thornton J.M. (1999). Motif-based searching in TOPS protein topology databases. Bioinformatics.

[bib0050] Grainger B., Sadowski M., Taylor W. (2010). Re-evaluating the “rules” of protein topology. J. Comput. Biol..

[bib0055] Greene L.H., Lewis T.E., Addou S., Cuff A., Dallman T., Dibley M., Redfern O., Pearl F., Nambudiry R., Reid A., Sillitoe I., Yeats C., Thornton J.M., Orengo C.A. (2007). The CATH domain structure database: new protocols and classification levels give a more comprehensive resource for exploring evolution. Nucleic Acids Res..

[bib0060] Grishin N. (2001). Fold change in evolution of protein structures. J. Struct. Biol..

[bib0065] Hadley C., Jones D.T. (1995). A systematic comparison of protein structure classifications SCOP, CATH and FSSP. Structure.

[bib0070] Harrison A., Pearl F., Mott R., Thornton J., Orengo C. (2002). Quantifying the similarities within fold space. J. Mol. Biol..

[bib0075] Harrison A., Pearl F., Sillitoe I., Slidel T., Mott R., Thornton J., Orengo C. (2003). Recognizing the fold of a protein structure. Bioinformatics.

[bib0080] Holm L., Sander C. (1993). Protein-structure comparison by alignment of distance matrices. J. Mol. Biol..

[bib0085] Hubbard T.J.P., Murzin A.G., Brenner S.E., Chothia C. (1997). SCOP: a structural classification of proteins database. Nucleic Acids Res..

[bib0090] Johannissen L.O., Taylor W.R. (2004). Protein fold comparison by the alignment of topological strings. Protein Eng..

[bib0095] Kolodny R., Petery D., Honig B. (2006). Protein structure comparison: implications for the nature of ‘fold space’, and structure and function prediction. Curr. Opin. Struct. Biol..

[bib0100] Krishna S., Grishin N. (2005). Structural drift: a possible path to protein fold change. Bioinformatics.

[bib0105] MacDonald J., Maksimiak K., Sadowski M., Taylor W. (2010). De novo backbone scaffolds for protein design. Protein Struct. Funct. Bioinform..

[bib0110] Maiorov V.N., Crippen G.M. (1994). Significance of root-mean-square deviation in comparing three-dimensional structures of globular proteins. J. Mol. Biol..

[bib0115] Murzin A.G., Brenner S.E., Hubbard T., Chothia C. (1995). SCOP: a structural classification of proteins database for the investigation of sequences and structures. J. Mol. Biol..

[bib0120] Orengo C.A., Flores T.P., Jones D.T., Taylor W.R., Thornton J.M. (1993). Recurring structural motifs in proteins with different functions. Curr. Biol..

[bib0125] Orengo C.A., Jones D.T., Thornton J.M. (1994). Protein superfaimiles and domain superfolds. Nature.

[bib0130] Orengo C.A., Michie A.D., Jones S., Jones D.T., Swindells M.B., Thornton J.M. (1997). CATH—a hierarchic classification of protein domain structures. Structure.

[bib0135] Orengo C.A., Thornton J.M. (1993). Alpha plus beta folds revisited: some favoured motifs. Structure.

[bib0140] Pan J.L., Bardwell J.C.A. (2006). the origami of thioredoxin-like folds. Protein Sci..

[bib0145] Peisajovich S., Rockah L., Tawfik D. (2006). Evolution of new protein topologies through multiestep gene rearrangements. Nat. Genet..

[bib0150] Petrey D., Fischer M., Honig B. (2009). Structural relationships among proteins with different global topologies and their implications for functional annotation strategies. Proc. Natl. Acad. Sci. U.S.A..

[bib0155] Petrey D., Honig B. (2009). Is protein classification necessary? Toward alternative approaches to function annotation. Curr. Opin. Struct. Biol..

[bib0160] Reeves G.A., Dallman T.J., Redfern O.C., Akpor A., Orengo C.A. (2006). structural diversity of domain superfamilies in the CATH database. J. Mol. Biol..

[bib0165] Reva B., Finkelstein A., Skolnick J. (1998). What is the probability of a chance prediction of a protein structure with an rmsd of 6 angstrom?. Folding Des..

[bib0170] Richardson J.S. (1977). β-Sheet topology and the relatedness of proteins. Nature.

[bib0175] Richardson J.S. (1985). Describing patterns of protein tertiary structure. Methods Enzymol..

[bib0180] Ruczinski I., Kooperberg C., Bonneau R., Baker D. (2002). Distributions of beta sheets in proteins with application to structure prediction. Protein Struct. Funct. Genet..

[bib0185] Sadowski M., Taylor W. (2010). On the evolutionary origins of “fold space continuity”: A study of topological convergence and divergence in mixed alpha-beta domains. J. Struct. Biol..

[bib0190] Sadreyev R., Kim B.-H., Grishin N. (2009). Discrete-continuous duality of protein structure space. Curr. Opin. Struct. Biol..

[bib0195] Salem G.M., Hutchinson E.G., Orengo C.A. (1999). Correlation of observed fold frequency with the occurrence of local structural motifs. J. Mol. Biol..

[bib0200] Sam V., Tai C., Garnier J., Gibrat J., Lee B., Munson P. (2006). Roc and confusion analysis of structure comparison methods identify the main causes of divergence from manual protein classification. BMC Bioinform..

[bib0205] Shindyalov I.N., Bourne P.E. (2001). An alternative view of protein fold space. Proteins Struct. Funct. Bioinform..

[bib0210] Sippl M. (2009). Fold space unlimited. Curr. Opin. Struct. Biol..

[bib0215] Skolnick J., Arakaki A., Lee S., Brylinski M. (2009). The continuity of protein structure space is an intrinsic property of proteins. Proc. Natl. Acad. Sci. U.S.A..

[bib0220] Taylor W., Chelliah V., Hollup S., Macdonald J., Jonassen I. (2009). Probing the dark matter of protein fold space. Structure.

[bib0225] Taylor W.R. (1999). Protein structure alignment using iterated double dynamic programming. Protein Sci..

[bib0230] Taylor W.R. (2002). A periodic table for protein structure. Nature.

[bib0235] Taylor W.R. (2006). Decoy models for protein structure score normalisation. J. Mol. Biol..

[bib0240] Taylor W.R. (2007). Evolutionary transitions in protein fold space. Curr. Opin. Struct. Biol..

[bib0245] Taylor W.R., Aszódi A. (2005). Protein Geometry, Classification, Topology and Symmetry.

[bib0250] Taylor W.R., Bartlett G.J., Chelliah V., Klose D., Lin K., Sheldon T., Jonassen I. (2008). Prediction of protein structure from ideal forms. Proteins Struct. Funct. Bioinform..

[bib0255] Taylor W.R., Lin K., Klose D., Fraternali F., Jonassen I. (2006). Dynamic domain threading. Proteins Struct. Funct. Bioinform..

[bib0260] Taylor W.R., Orengo C.A. (1989). Protein structure alignment. J. Mol. Biol..

[bib0265] Taylor W.R., Thornton J.M. (1984). Recognition of super-secondary structure in proteins. J. Mol. Biol..

[bib0270] Theobald D.L., Wuttke D.S. (2006). divergent evolution within protein superfolds inferred from profile-based phylogenetics. J. Mol. Biol..

[bib0275] Valas R., Yang S., Bourne P. (2009). Nothing about protein structure classification makes sense except in the light of evolution. Curr. Opin. Struct. Biol..

[bib0280] Westhead D., Hatton D., Thornton J. (1998). An atlas of protein topology cartoons available on the world-wide web. Trends Biochem. Sci..

[bib0285] Wrabl J., Grisin N.V. (2008). Statistics of random protein superpositions: p-values for pairwise structure alignment. J. Comput. Biol..

[bib0290] Xu J., Zhang Y. (2010). How significant is a protein structure similarity with TM-score = 0.5?. Bioinformatics.

[bib0295] Zhang Y., Skolnick J. (2004). Scoring function for automated assessment of protein structure template quality. Protein Struct. Funct. Bioinform..

[bib0300] Zhang Y., Skolnick J. (2005). Tm-align: a protein structure alignment algorithm based on the TM-score. Nucleic Acids Res..

